# B7-H4 Immune Checkpoint Protein Affects Viability and Targeted Therapy of Renal Cancer Cells

**DOI:** 10.3390/cells11091448

**Published:** 2022-04-25

**Authors:** Maite Emaldi, Caroline E. Nunes-Xavier

**Affiliations:** 1 Biomarkers in Cancer Unit, Biocruces Bizkaia Health Research Institute, Plaza de Cruces 12, 48903 Barakaldo, Spain; maite.emaldimartinezdeilarduya@osakidetza.eus; 2 Department of Tumor Biology, Institute for Cancer Research, Oslo University Hospital, The Norwegian Radium Hospital, 0310 Oslo, Norway

**Keywords:** B7-H4, immune checkpoint protein, renal cancer cells, targeted therapies, tyrosine kinase inhibitors, mTOR inhibitors

## Abstract

Targeted therapy in combination with immune checkpoint inhibitors has been recently implemented in advanced or metastatic renal cancer treatment. However, many treated patients either do not respond or develop resistance to therapy, making alternative immune checkpoint-based immunotherapies of potential clinical benefit for specific groups of patients. In this study, we analyzed the global expression of B7 immune checkpoint family members (PD-L1, PD-L2, B7-H2, B7-H3, B7-H4, B7-H5, B7-H6, and B7-H7) in human renal cancer cells (Caki-1, A-498, and 786-O cell lines) upon treatment with clinically relevant targeted drugs, including tyrosine kinase inhibitors (Axitinib, Cabozantinib, and Lenvatinib) and mTOR inhibitors (Everolimus and Temsirolimus). Gene expression analysis by quantitative PCR revealed differential expression patterns of the B7 family members in renal cancer cell lines upon targeted drug treatments. B7-H4 gene expression was upregulated after treatment with various targeted drugs in Caki-1 and 786-O renal cancer cells. Knocking down the expression of B7-H4 by RNA interference (RNAi) using small interfering RNA (siRNA) decreased renal cancer cell viability and increased drug sensitivity. Our results suggest that B7-H4 expression is induced upon targeted therapy in renal cancer cells and highlight B7-H4 as an actionable immune checkpoint protein in combination with targeted therapy in advanced renal cancer cases resistant to current treatments.

## 1. Introduction

Renal cell carcinoma refers to the heterogeneous group of cancers derived from renal tubular epithelial cells and constitutes one of the ten most common cancers worldwide. Clear cell renal cell carcinoma (ccRCC) is one of the major subtypes of renal cancer and represents most kidney-cancer-derived deaths [1]. Besides ccRCC, other types of renal carcinoma have also been histologically classified, such as papillary and chromophobe renal cell carcinomas. Localized renal cell carcinomas are usually treated with nephron-sparing surgery, also known as partial nephrectomy, where the malignant tissue in the kidney is removed [2]. Some localized renal cell carcinoma cases can also be treated with radical nephrectomy with complete removal of the kidney. However, around 30% of the patients with localized ccRCC treated with nephrectomy finally develop metastases, which are associated with high mortality [2]. Radiotherapy and chemotherapy are not frequently used to treat this type of cancer since they have been considered mostly ineffective for patients with renal cell carcinoma. Besides surgery, the current therapeutic landscape of renal cell carcinoma mainly consists of targeted therapy and immunotherapy [3]. Current major immunotherapies in cancer, including renal cancer, are based on the blocking by specific monoclonal antibodies of the binding of the B7 immune checkpoint proteins CD80/B7-1 and CD86/B7-2, and PD-L1/B7-H1 and PD-L2/B7-DC, with their co-receptors CTLA-4 and PD-1, respectively. This results in the blockage of the inhibitory effect of the immune checkpoints on T cells, enabling them to restore the antitumor activity [4,5].

The introduction of tyrosine kinase inhibitors (TKI) and mTOR targeted therapies in combination with immunotherapies has been a major step forward in the treatment of renal cell carcinoma [1,3]. Nevertheless, administered alone, they hardly induce a complete response, and they have not been able to induce permanent disease remission [6]. Tumors developing resistance are believed to carry out an “angiogenic switch” by changes in the gene expression so that more molecules involved in tumor progression are expressed [7]. Nevertheless, precise mechanisms of resistance to immunotherapies and targeted therapies in renal cancer remain unclear. To better understand the biology of tumorigenesis and metastasis in the clinical context, elucidating the mechanisms of resistance to targeted therapies is of great importance.

Proteins belonging to the B7 family of immunoregulatory proteins are highly expressed in a variety of cancers, correlating with cancer progression and poor prognosis [8,9]. Several B7 family members are overexpressed in renal cell carcinoma in correlation with tumor immune evasion, increased disease progression, and decreased survival [9]. Previous studies have shown that the expression of B7 family members, such as PD-L1, B7-H3, B7-H4, and B7-H6, is associated with poor outcomes in patients with renal cell carcinoma [10,11]. Since aberrant expression of B7 proteins has been reported to correlate with the progression of renal cell carcinoma, these proteins could be used as markers for predicting tumor development and resistance to therapy.

B7-H4 protein expression is limited in normal tissues, but aberrant B7-H4 expression is found in different solid tumor types, including breast [12], serious ovarian [13], colorectal [14] and pancreatic [15], lung [16], prostate [17], and renal cancer [18], as well as hematological malignancies including myeloma [19]. In renal cell carcinoma, high B7-H4 expression is present in both tumor cell and tumor vasculature endothelial cells, which positively correlates with tumor progression, whereas low expression was observed in normal renal endothelial cells [18,20]. Moreover, B7-H4-positive tumors seem to be more aggressive and associated with increased risk for disease progression and decreased patient survival. Enhanced tumor aggressiveness is a consequence of the activity of B7-H4 as a negative mediator of T cells. In addition, B7-H4 can induce the unresponsiveness of tumor cells to apoptosis [21]. Evidence suggests that B7-H4 might participate in renal cell carcinoma tumorigenesis by enabling tumor neovascularization at sites relatively distant from the tumor cells [18]. Serum soluble B7-H4 was found to be associated with poor outcomes and overall survival of renal cancer patients, and it has been proposed for predicting the prognosis of patients with non-metastatic clear cell renal cell carcinoma [22]. In normal tumor-adjacent kidney specimens, B7-H4 was found either absent or with a focal and sporadic membranous immunostaining in distal convoluted renal tubules [18]. B7-H4 has been identified in renal cells as a membrane protein but also as a cytoplasmic-nuclear shuttling protein [20]. The coding sequence of the protein contains a signal peptide and a nuclear localization sequence (NLS), and upon inhibition of nuclear export by Leptomycin B, B7-H4 accumulated in the nucleus [20]. Thus, B7-H4 protein can shuttle between membrane, cytoplasm, and nucleus [20]. In addition, it has also been postulated B7-H4 anchorage to the membrane by glycophosphatidylinositol linkage [23]. Nuclear and membrane localizations of the protein have been associated with disease progression, and membrane localization was found to be inversely correlated with the presence of tumor-infiltrating lymphocytes [20].

The hypothesis of this work is that alternative members of the B7 family of immune checkpoint proteins could be actionable targets in combination with currently approved targeted therapy in renal cancer cells. In this study, we analyzed the global gene expression profile of B7 family members, including PD-L1, PD-L2, B7-H2, B7-H3, B7-H4, B7-H5, B7-H6, and B7-H7 in renal cancer cells upon treatment with different targeted therapies, and we tested by functional in vitro experiments the effect of B7 expression on the sensitivity to targeted therapies in renal cancer cells.

## 2. Materials and Methods

### 2.1. Media and Cell Growth

Caki-1 cells were maintained in McCoy’s 5A (Lonza, Basel, Switzerland) medium, 786-O cells were maintained in RPMI 1640 (Lonza) medium, A-498 cells were cultured in EMEM (Minimum Essential Medium Eagle, Lonza) medium, and HEK293 cells were cultured in DMEM (Dulbecco’s Modified Eagle’s Medium, Lonza) medium. All media were supplemented with 10% FBS (Fetal Bovine Serum, Sigma-Aldrich, St. Louis, MO, USA), 1% L-Glutamine (Sigma-Aldrich), and 1% penicillin/streptomycin (Sigma-Aldrich). Cells were incubated at 37 °C and 5% CO_2_.

### 2.2. Targeted Therapy Treatment, Cell Viability, and Proliferation Assays

To measure cell viability, 1500 HEK293 cells, 3000 Caki-1 cells, 1500 786-O, or A-498 cells were plated per well in 96-well culture plates. A day after plating the cells, different concentrations of the drugs (Axitinib, Cabozantinib, Lenvatinib, Everolimus, or Temsirolimus) or vehicle (DMSO, Sigma-Aldrich) were added. HEK293 cells were treated with 1 μM Axitinib, Cabozantinib, and Lenvatinib. Renal cancer cells were treated with 20 μM Axitinib, 8 μM Cabozantinib, and 20 μM Lenvatinib. All cells were treated with 0.1 μM Everolimus and 0.1 μM Temsirolimus. All inhibitors were from Selleckchem (Houston, TX, USA). Cells were incubated for 72 h, and cell proliferation was determined by crystal violet assay. Then, 50 μL of 0.5% crystal violet (Sigma-Aldrich) was added to each well for 20 min after cells had been washed with PBS (Phosphate Buffered Saline, Lonza) and fixed in 70% Ethanol (Merck Millipore, Burlington, MS, USA) for 10 min at −20 ℃. Absorbance was measured at 560 nm using iMark™ Microplate Absorbance Reader (Bio-Rad, Hercules, CA, USA). Cell viability is presented relative to the corresponding negative control of each cell type.

Cell proliferation was measured with the CellTiter 96^®^AQ_ueous_ One Solution Cell Proliferation Assay Kit (MTS Assay, Promega, Madison, WI, USA) in 96-well plates. All the cells were plated at the same densities and drug concentrations as in the crystal violet assay. Cell viability was measured after 72 h of treatment; 20 μL of the CellTiter reagent was added to each well for 90 min, and absorbance was measured at 490 nm using Mark™ Microplate Absorbance Reader (Bio-Rad). Cell proliferation is presented as the percentage with respect to the corresponding negative control.

### 2.3. RNA Isolation, Reverse Transcription, and Quantitative PCR

Real-time quantitative PCR (RT-qPCR) was performed to analyze B7 gene expression. Caki-1, 786-O cells, and A-498 were treated with 10 μM Axitinib, 5 μM Cabozantinib, 10 μM Lenvatinib, 0.1 μM Everolimus, 0.1 μM Temsirolimus, or DMSO (control) for 24 h. Total mRNA was extracted with the Illustra™ RNAspin Mini RNA Isolation Kit following the manufacturer’s protocol (GE Healthcare Life Sciences, Marlborough, MS, USA). Subsequently, 1 μg of total RNA was used for cDNA synthesis following the Thermo Scientific RevertAid Reverse Transcriptase protocol (ThermoFisher, Waltham, MS, USA). RT-qPCR reactions were carried out as previously described [24], using Agilent AriaMx Real-Time PCR System (Agilent Technologies, Santa Clara, CA, USA). Primers used were: QuantiTect Primers (Qiagen, Germantown, MD, USA) for PD-L1 (QT00082775, official gene name *CD274*), PD-L2 (QT00089761, official gene name *PDCD1LG2*), B7-H2 (QT00004669, official gene name *ICOSLG*), B7-H3 (QT00013608, official gene name *CD276*), B7-H4 (QT01025584, official gene name *VTCN1*), B7-H5 (QT01024597, official gene name *VSIR*), B7-H6 (QT00075971, official gene name *NCR3LG1*), and B7-H7 (QT00197092, official gene name *HHLA2*). Finally, relative fold change was calculated using the 2^−ΔΔCt^ equation using *HPRT* as housekeeping gene. Gene expression changes are represented as relative fold changes in logarithmic scale with base 2 (Log_2_).

### 2.4. Plasmids, Site-Directed Mutagenesis, DNA Extraction, Quantification, and Sequencing

B7-H4 cDNA open reading frame clone NM_024626.3, cloned in pcDNA3.1^+^/C-DYK, was purchased from Genescript (Piscataway, NJ, USA). Site-directed mutagenesis was performed to remove the C terminal Flag tag from the B7-H4 sequence. The process was carried out by one-step inverse PCR, as described in reference [25], using oligos 5′- CCT TAC CTG ATG CTA AAA TGA TAA ACC CGC TGA TCA -3′ (forward), and 5′-TGA TCA GCG GGT TTA TCA TTT TAG CAT CAG GTA AGG-3′ (reverse). Plasmid DNA was extracted from *E. coli* following the NucleoSpin^®^ Plasmid EasyPure kit procedure (Nacherey-Nagel, Düren, Germany). Sequences were verified at the Genetic and Genomic Core facility from Biocruces Bizkaia Health Research Institute.

### 2.5. Transient Transfections

GenJet™ protocol was used for B7-H4 overexpression in the HEK293 cell line (Signagen, Frederick, MD, USA). HEK293 cells were plated in a 6-well plate for Western blot analysis or in an 8-well chamber slide for immunofluorescence assay. Lipofectamine^®^ RNAiMAX Reagent protocol (ThermoFisher) was used for B7-H4 interference (RNAi) by short interfering RNAs (siRNAs) in Caki-1 and 786-O cell lines. Cells were plated in 6-well plates for Western blot analysis or in 96-well plates for posterior viability assay; 24 h after plating the cells, the silencing was performed following the RNAiMAX transfection procedure using 20 nM siRNAs. siRNAs for the human B7-H4 gene used were from FlexiTube GeneSolution (GS79679 for VTCN1/B7-H4, Product number: 1027416; SI04365039 (siB7-H4 #1) and SI04346433 (siB7-H4 #2), FlexiTube siRNA Qiagen), and si non-specific (siNS) RNAs and si GAPDH (AM4605; ThermoFisher).

### 2.6. Cell Lysis and Western Blot

Cells were lysed in M-PER lysis buffer (ThermoFisher) and processed for Western blot as described in [26]. Primary antibodies used were rabbit anti-B7-H4 (1:500, D1M81, Cell Signaling, Danvers, MA, USA), mouse anti-Flag (1:500, MAB3118, Sigma-Aldrich), mouse anti-GAPDH (1:500, 6C5, Santa Cruz Technology, Dallas, TX, USA), and mouse anti-α-Tubulin (1:200, B-7, Santa Cruz Technology). Secondary antibodies were IRDye 680RD and 800CW Goat anti-Mouse and Goat anti-Rabbit (LI-COR, Lincoln, NE, USA). Odyssey CLx (Li-Cor^®^) Image Studio v4.0.21 software was used to visualize fluorescence signals on the membranes.

### 2.7. Immunofluorescence Assay

First, 3 × 10^4^ HEK293 cells/well were plated in 8-well chamber slides for immunofluorescence (Ibidi, Gräfelfing, Germany). Transient transfection was performed as described above, and cells were washed and fixed in Methanol (Sigma-Aldrich) for 5 min at −20 °C and blocked in blocking solution (Phosphate Buffered Saline (PBS, Sigma-Aldrich) containing 3% Bovine Serum Albumin (BSA, Sigma-Aldrich). Rabbit anti-B7-H4 primary antibody (1/200 in blocking solution) was incubated overnight at 4 ℃ in a wet chamber. Subsequently, cells were washed three times with PBS-BSA for 10 min prior to incubation with anti-rabbit FITC secondary antibody (1/100) for 1 h in a wet chamber and darkness at room temperature. Cells were washed and mounted in a Mounting Medium with DAPI (Abcam, Cambridge, UK) and visualized in a confocal microscope (ZEISS LSM880 Airyscan, Jena, Germany). For quantitation of B7-H4 subcellular distribution, at least 50 positive cells were scored. Cells were rated as membrane staining (M) or membrane/cytoplasm (M/C). Nuclei were identified by DAPI staining.

### 2.8. Statistical Analysis

Error bars in results represent ± standard deviation (S.D.). Data were analyzed by GraphPad Prism *t* Test Calculator, where significance was calculated using a two-tailed student *t*-test. Subcellular localization was analyzed by the Chi-square test. *p* values smaller than 0.05 were considered significant and are indicated with an asterisk (*). All experiments were performed at least twice, and the results shown are from one representative experiment.

## 3. Results

### 3.1. Treatment with Tyrosine Kinase Inhibitors (TKI) or with mTOR Inhibitors Decreases Renal Cancer Cell Viability

**Figure 1:**
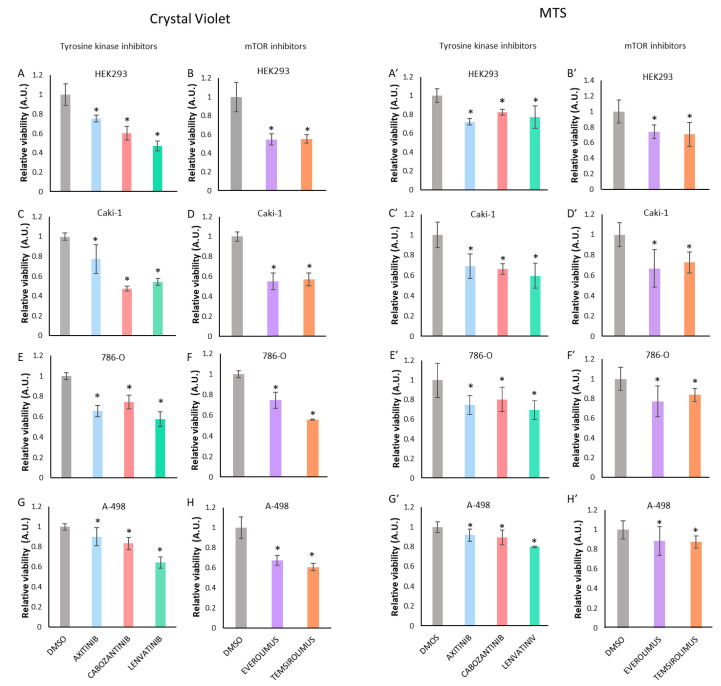
Proliferation of HEK293, Caki-1, 786-O, and A-498 cells upon treatment with tyrosine kinase inhibitors or with mTOR inhibitors. Crystal violet (CV) staining (**A**–**H**) and MTS assays (**A**’–**H**’) were used to measure viability of cells upon treatment with tyrosine kinase and mTOR inhibitors after 72 h. Concentrations used were 1 μΜ Axitinib, 1 μΜ Cabozantinib, and 1 μΜ Lenvatinib in HEK293 cells (**A**,**A**’); and 20 μΜ Axitinib, 8 μΜ Cabozantinib, and 20 μΜ Lenvatinib in Caki-1 (**C**,**C**’), 786-O (**E**,**E**’), and A-498 (**G**,**G**’) cells. Everolimus and Temsirolimus were used at 0.1 μΜ in all cells (**B**,**B**’,**D**,**D**’,**F**,**F**’,**H**,**H**’). Note that HEK293 cells were more sensitive to the TKI treatments than the other renal cancer cells. Data are shown as relative proliferation ± S.D. Statistically significant results (*p* < 0.05) are marked with *. All data were normalized relative to untreated cells and are shown in arbitrary units (A.U.).

To study the effect of currently used TKI and mTOR inhibitors in renal cancer treatment, HEK293, Caki-1, 786-O, and A-498 cell lines were treated with three TKI (Axitinib, Cabozantinib, and Lenvatinib) or with two mTOR inhibitors (Everolimus and Temsirolimus). In the four cell lines used, treatment with both tyrosine kinase and mTOR inhibitors resulted in a significant decrease in cell viability (Figure 1). The dose and time of the inhibitor’s treatments were chosen based on time-course experiments of both cell viability and proliferation (Supplementary Figure S1) and dose–response experiments (Supplementary Figure S2).

### 3.2. B7-H4 Expression Is Increased upon Treatment with Tyrosine Kinase Inhibitors or with mTOR Inhibitors

**Figure 2:**
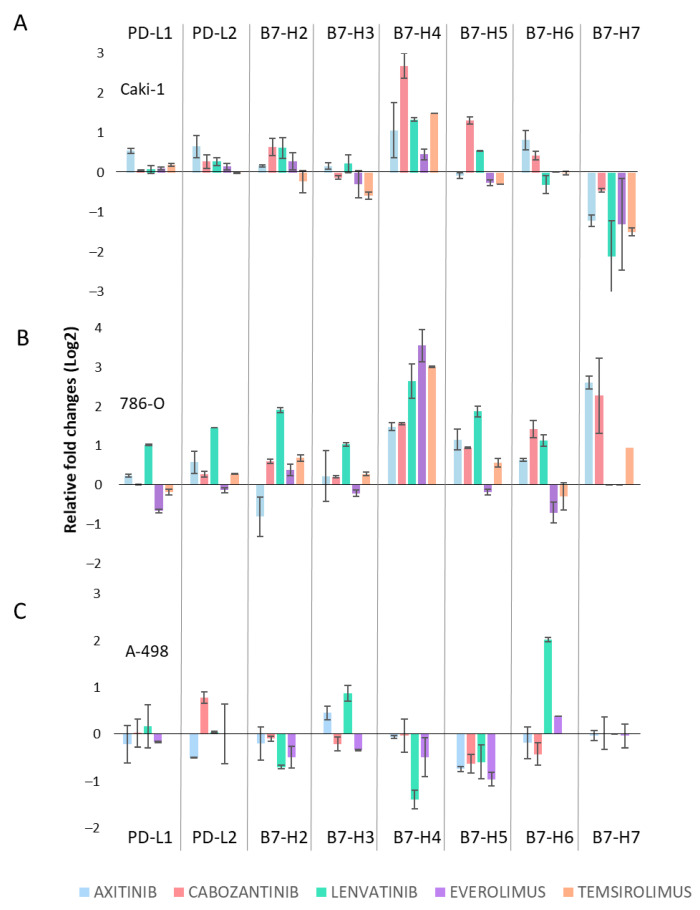
B7 family gene expression upon treatment with tyrosine kinase inhibitors or with mTOR inhibitors. B7 family gene expression in Caki-1 (**A**), 786-O (**B**), and A-498 (**C**) cells upon treatment with tyrosine kinase and mTOR inhibitors was measured by real-time quantitative PCR (RT-qPCR). Relative fold changes of PD-L1, PD-L2, B7-H2, B7-H3, B7-H4, B7-H5, B7-H6, and B7-H7 mRNA expression are represented in a logarithmic scale (Log_2_). Cells were treated for 24 h with tyrosine kinase inhibitors (10 μM Axitinib, 5 μM Cabozantinib, and 10 μM Lenvatinib) and mTOR inhibitors (0.1 μM Everolimus and 0.1 μM Temsirolimus).

To analyze the global gene expression profile of the B7 family, RT-qPCR was performed in Caki-1, 786-O, and A-498 cell lines after 24 h treatment with TKI (10 μM Axitinib, 5 μM Cabozantinib, 10 μM Lenvatinib) or with mTOR (0.1 μM Everolimus and 0.1 μM Temsirolimus) inhibitors. For gene expression analysis, we chose a shorter time and lower concentrations of the TKI inhibitors to avoid secondary effects of cell death upon longer incubations (Supplementary Figures S1 and S2). Interestingly, the B7-H4 gene was upregulated in Caki-1 and 786-O cells 24 h after treatment with several of the different drugs employed (Figure 2A,B). On the other hand, B7-H4 expression was not consistently increased in A-498 cells (Figure 2C). In some cases (such as B7-H5 in Caki-1 cells and B7-H6 in 786-O cells), we saw opposing effects of TKI and mTOR inhibitors. In other cases (for instance, B7-H6 in A498 cells), changes in gene expression were only observed upon treatment with some inhibitors.

### 3.3. Membrane Localization of B7-H4 in Renal Cells

**Figure 3:**
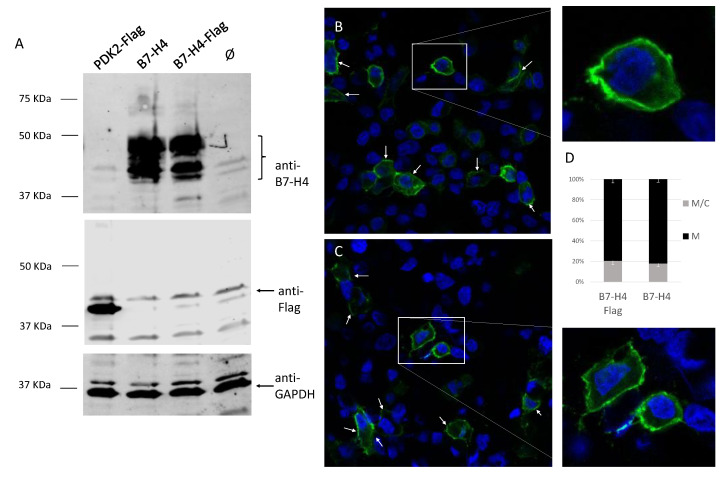
Ectopic overexpression of B7-H4 in HEK293 cells. (**A**) HEK293 cells were transfected with pCDNA3.1 empty vector (Ø) or containing B7-H4 or B7-H4-Flag and processed for Western blot using anti-B7-H4, anti-Flag, and anti-GAPDH antibodies. PDK2-Flag was used as a control for Flag antibody. Molecular weight markers are indicated on the left. (**B**,**C**) Subcellular localization of B7-H4 and B7-H4-Flag in HEK293 cells. HEK293 cells were transfected to overexpress B7-H4 (**B**) or B7-H4-Flag tag (**C**) and processed for immunofluorescence using anti-B7-H4 antibodies. Nuclei are shown in blue (DAPI staining), and B7-H4 proteins are shown in green (FITC). White arrows highlight membrane immunostaining patterns. Images were taken at 63X. (**D**) Subcellular localization of B7-H4-Flag and B7-H4. Data are shown as the percentage of cells displaying membrane (M) or membrane/cytoplasmic (M/C) localization.

B7-H4 has been proposed to be anchored to the plasma membrane through glycosyl phosphatidylinositol (GPI) linkage [23]. To test the expression and subcellular localization of B7-H4 ectopic overexpression in renal cancer cells, ectopic overexpression of B7-H4 with and without Flag epitope was carried out in HEK293 cells, transfected with pcDNA3.1 vector containing B7-H4 or B7-H4-Flag. Overexpression was visualized by Western blot (WB) analysis using anti-Flag and anti-B7-H4 antibodies (Figure 3A). We did not detect B7-H4-Flag by WB with an anti-Flag antibody (Figure 3A). However, using an anti-B7-H4 antibody, we detected B7-H4 in both B7-H4-Flag- and B7-H4-transfected cells, indicating that the Flag epitope might be cleaved from B7-H4 during biogenesis.

Next, subcellular localization of B7-H4 with and without Flag epitope was tested in HEK293 cells. Immunofluorescence analysis using an anti-B7-H4 antibody showed no difference in the subcellular localization of B7-H4 regardless of the Flag epitope (*p* = 0.3029). In both cases, B7-H4 showed mainly membrane distribution (Figure 3B–D). The lack of detection of the Flag epitope and B7-H4 membrane expression goes in line with the proposed B7-H4 GPI anchoring to the membrane [23].

### 3.4. B7-H4 Silencing and Tyrosine Kinase and mTOR Inhibitors Affect Viability of Renal Cancer Cells

**Figure 4:**
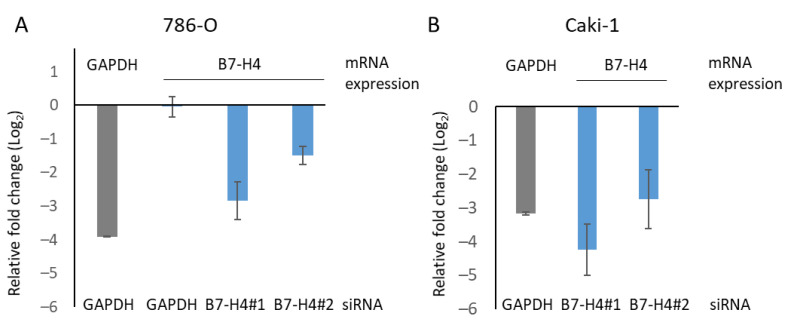
B7-H4 silencing analysis by RT-qPCR in renal cancer cells. Relative fold change in mRNA expression of GAPDH and B7-H4 by RT-qPCR of 786-O (**A**) and Caki-1 (**B**) cells. siRNA used are indicated in the bottom: GAPDH (siGAPDH transfected cells, positive control), B7-H4 #1 and B7-H4 #2 (siB7-H4 #1 and #2 transfected cells).

The role of B7-H4 on drug sensitivity of renal cancer cells was studied by measuring cell proliferation of B7-H4-silenced (siB7-H4) and non-specific-silenced (siNS) control cells upon treatment with targeted therapy. Two different B7-H4 siRNAs were transiently transfected in Caki-1 and 786-O cells, and GAPDH was also silenced as a control. To test the efficacy of the different siRNAs, silencing was monitored by RT-qPCR (Figure 4). In our experimental setting, we were not able to detect endogenous B7-H4 protein expression by Western blot, and B7-H4 silencing was confirmed by RT-qPCR. As shown, siGAPDH was efficient for GAPDH silencing, and siB7-H4 #1 and siB7-H4 #2 were efficient for the silencing of B7-H4 (Figure 4) and were chosen for functional silencing experiments in Caki-1 and 786-O cells.

**Figure 5:**
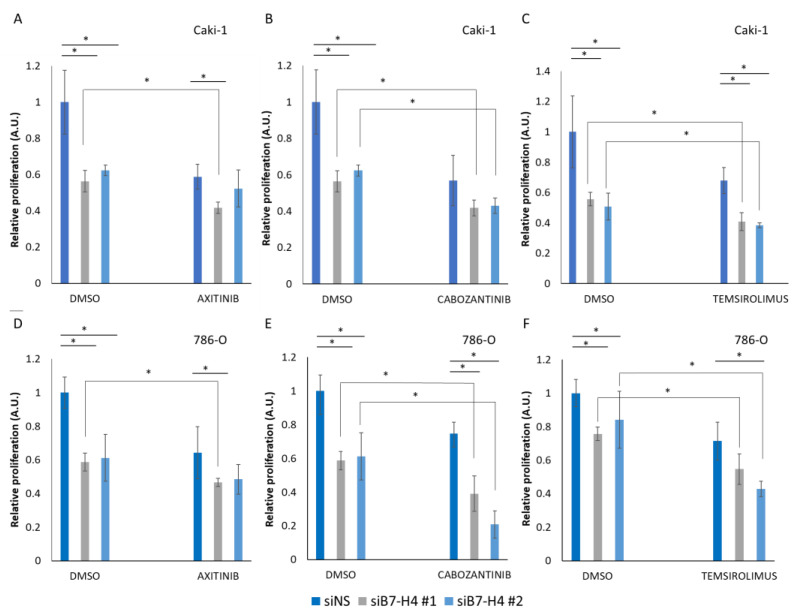
Proliferation analysis of Caki-1 and 786-O renal cancer cells after B7-H4 silencing and treatment with tyrosine kinase and mTOR inhibitors. MTS assay was used to measure proliferation of siB7-H4 Caki-1 (**A**–**C**) and 786-O (**D**–**F**) cells after 72 h of treatment with vehicle (DMSO), 20 μM Axitinib (**A**,**D**), 8 μM Cabozantinib (**B**,**E**), or 0.1 μM Temsirolimus (**C**,**F**). Data are shown as relative proliferation ± S.D. Statistically significant results (*p* < 0.05) are marked with *. All data were normalized relative to untreated siNS cells and are shown in arbitrary units (A.U.).

Caki-1 and 786-O cells were transfected for B7-H4 silencing with siB7-H4 #1 and siB7-H4 #2 or with siNS as control. Afterward, they were treated with TKI or with mTOR inhibitors, and cell proliferation was measured by MTS assay 72 h after treatment. Silencing of B7-H4 significantly inhibited cell proliferation in both Caki-1 and 786-O cells. Moreover, an additive effect on cell growth inhibition was seen when siB7-H4 transfected cells were treated with the tested drugs (Figure 5). This suggests a role for B7-H4 in renal cancer cell growth and that B7-H4 is an actionable target in combination with targeted therapies.

## 4. Discussion

Several B7 family members, including B7-H4, are overexpressed in renal cell carcinoma, in correlation with increased disease progression and decreased patient survival [27]. Radiotherapy and chemotherapy have been considered ineffective for renal cell carcinoma treatment, but the introduction of TKI and mTOR targeted therapies and immunotherapies in combinations has increased the response rate of patients [3]. However, many renal cell carcinoma patients are either non-responsive to the treatment from the beginning or acquire resistance during the treatment. The global aim of this work was to test the involvement of B7 family members in sensitivity to currently approved targeted therapy in renal cancer cells.

An increase in B7-H4 gene expression was consistently observed in Caki-1 and 786-O cell lines upon treatment with TKI and mTOR inhibitors, but not in A-498 cells (Figure 2). This could indicate that B7-H4 plays a role in the drug response of renal cancer cells. The different B7 expression profile in A-498 cells could be related to the recent findings suggesting that these cells might be of papillary origin [28]. The possibility that A-498 cells display unique gene expression regulatory elements when compared to Caki-1 or 786-O cells deserves further studies. We additionally observed small changes in gene expression in other B7 genes, as well as some opposing effects upon treatment with TKI or mTOR inhibitors.

Changes in B7-H7 expression were also detected upon treatment with TKI and mTOR inhibitors. B7-H7 expression was downregulated in Caki-1 cells and increased in 786-O cells (Figure 2). Increased B7-H7 expression has been observed in ccRCC, associated with poor outcome and tumor progression, and has been suggested as a biomarker for renal cell carcinoma [22]. Downregulation of B7-H7 after treatment with targeted therapy could indicate that B7-H7 might be involved in cell proliferation. Further studies are required to assess this hypothesis. We also observed opposing effects in some B7 gene expression patterns upon different drug treatments, which could be due to differences in the specificity of the inhibitors. Axitinib is a highly selective inhibitor of VEGFR that competitively binds to the ATP-binding site of the kinase. Lenvatinib also binds to the ATP-binding site of kinases, but unlike Axitinib, Lenvatinib is a multitargeted TKI, and its antitumor and antiangiogenic activities are carried out via inhibition of VEGFR, fibroblast growth factor receptor (FGFR), ret proto-oncogene kinase receptor (RET), and platelet-derived growth factor receptor (PDGFR). Cabozantinib is a strong inhibitor of VEGFR but also targets MET tyrosine kinase. These differences in the specificity of the TKI could explain the opposing effects seen in the qPCR analysis.

Upregulation of B7-H4 has been reported in hypoxia-associated pathological situations in multiple myeloma cells, and overexpression of HIF-1α was associated with increased transcription of B7-H4 [19]. It is possible that HIF-1α mediates B7-H4 induced expression in renal cancer cell lines upon treatment with targeted therapies. In renal cell carcinoma, B7-H4 expression has been detected in tumor cells and in tumor vasculature endothelial cells, and patients with B7-H4-expressing renal cell carcinoma tumors were more likely to die from the disease compared to patients with B7-H4-negative tumors [18].

At the subcellular level, B7-H4 protein has been reported to be expressed on the plasma membrane, in the cytosol, and in the nucleus of renal cancer cells, as it has been identified as a membrane/cytoplasmic-nuclear shuttling protein with a nuclear localization sequence (NLS) [20]. Both membrane and nuclear localization of the protein have been associated with disease progression, and membrane localization has specifically been shown to have an effect on cell proliferation [20]. In our immunofluorescence experiments, B7-H4 protein displayed mostly a membrane localization, and no protein was detected in the nucleus (Figure 3). Unlike other B7 family members, B7-H4 has only two amino acids predicted to be located in the cytosolic portion, and it has been proposed that B7-H4 is anchored to the membrane by glycophosphatidylinositol linkage [23]. This might affect B7-H4 accumulation at the cell surface and limit B7-H4 detection in a cell-specific manner. Using C-terminal tagged B7-H4-Flag, we have observed that the Flag epitope is lost upon B7-H4-Flag ectopic expression, in line with GPI anchorage. Whether or not B7-H4 expression in the plasma membrane in our conditions is dependent on GPI requires further experimental work.

Proliferation assay results on Caki-1 cells treated with TKI and mTOR inhibitors showed that B7-H4 silencing had an additive effect on sensitivity to Axitinib, Cabozantinib, and Temsirolimus (Figure 5). Consistently, B7-H4 expression was upregulated in Caki-1 cells treated with TKI and mTOR inhibitors (Figure 2). These results suggest an independent downregulation of cell viability by TKI/mTOR inhibitors or by siRNA targeting B7-H4 in renal cancer cells. It has been documented that B7-H4 has a tumor-promoting role in addition to T-cell inhibition [17], and it was shown that overexpression of B7-H4 in Caki-1 cells led to less sensitivity to the chemotherapeutic agents doxorubicin or docetaxel [20]. Additionally, silencing B7-H4 improved the effectiveness of chemotherapy in breast cancer, reinforcing its role in chemoresistance [29]. Consistently, blocking B7-H4 in breast cancer is synergistic with PD-1 blockage [30], trastuzumab treatment [31], and doxorubicin, paclitaxel, and carboplatin [29], suggesting it is widely involved in resistance to anti-cancer therapies. In this regard, a clinical trial using an anti-B7-H4 monoclonal antibody in combination with pembrolizumab is currently ongoing for advanced solid tumors (ClinicalTrials.gov Identifier: NCT03514121), which will be important to assess the safety and efficacy in patients, including the effect of these combinatory therapies on normal tissues, including normal renal cells. Other B7 proteins, including PD-L1/B7-H1, B7-H3, and B7-H6, have been involved in drug sensitivity to chemotherapies and targeted therapies in several cancer cell types [8,32,33,34,35,36]. This highlights the potential of B7 family members as attractive combinatory anti-cancer targets against therapy-resistant cancers.

## 5. Conclusions

In conclusion, our findings support the notion that B7-H4 may play a key role in renal cell carcinoma growth and is induced in renal cancer cells upon treatment with targeted therapy. Targeting B7-H4 could be beneficial in combination with targeted therapy in renal cell carcinoma treatment and ultimately improve patient survival. A dedicated analysis is required to further characterize the role of B7-H4 in renal cancer cell drug sensitivity.

## Data Availability

Not applicable.

## Supplementary Materials

The following supporting information can be downloaded at: https://www.mdpi.com/article/10.3390/cells11091448/s1, Figure S1: Proliferation of HEK293, Caki-1, 786-O, and A-498 cells upon time-course treatment with tyrosine kinase inhibitors or with mTOR inhibitors; Figure S2: Proliferation of HEK293, Caki-1, 786-O, and A-498 cells upon treatment with different doses of tyrosine kinase inhibitors or with mTOR inhibitors.
