# Supplementary material for: B7-H4 Immune Checkpoint Protein Affects Viability and Targeted Therapy of Renal Cancer Cells
Source: Cells. 2022 Apr 25;11(9):1448. doi: 10.3390/cells11091448 (PMC9104196; doi:10.3390/cells11091448)

Supplementary Figure legends

Supplementary Figure S1.

Proliferation of HEK293, Caki-1, 786-O and A-498 cells upon treatment with tyrosine kinase inhibitors or with mTOR inhibitors. Cristal violet staining and MTS assays were used to measure viability of cells upon treatment with tyrosine kinase and mTOR inhibitors after 24, 48 and 72 h. Concentrations used were 1 µM Axitinib, 1 µM Cabozantinib and 1 µM Lenvatinib in HEK293 cells; and 20 µM Axitinib, 8 µM Cabozantinib, and 20 µM Lenvatinib in Caki-1, 786-O and A-498 cells. Everolimus and Temsirolimus were used at 0.1 µM in all cells. Note that HEK293 cells were more sensitive to the TKI treatments than the other renal cancer cells. Data is shown as relative proliferation ±S.D. All data were normalized relative to untreated cells and are shown in arbitrary units (A.U.).

Supplementary Figure S2.

Proliferation of HEK293, Caki-1, 786-O and A-498 cells upon treatment with different doses of tyrosine kinase inhibitors or with mTOR inhibitors. Cristal violet staining was used to measure viability of cells upon treatment with tyrosine kinase and mTOR inhibitors after 72 h, and the concentrations used were 1, 10 and 20 µM Axitinib; 1, 5 and 8 µM Cabozantinib; 1, 10 and 20 µM Lenvatinib; 0.1, 1 and 10 µM Everolimus and 0.1, 1 and 10 µM Temsirolimus. Data is shown as relative proliferation ±S.D. All data were normalized relative to untreated cells and are shown in arbitrary units (A.U.).

Supplementary Figure S1

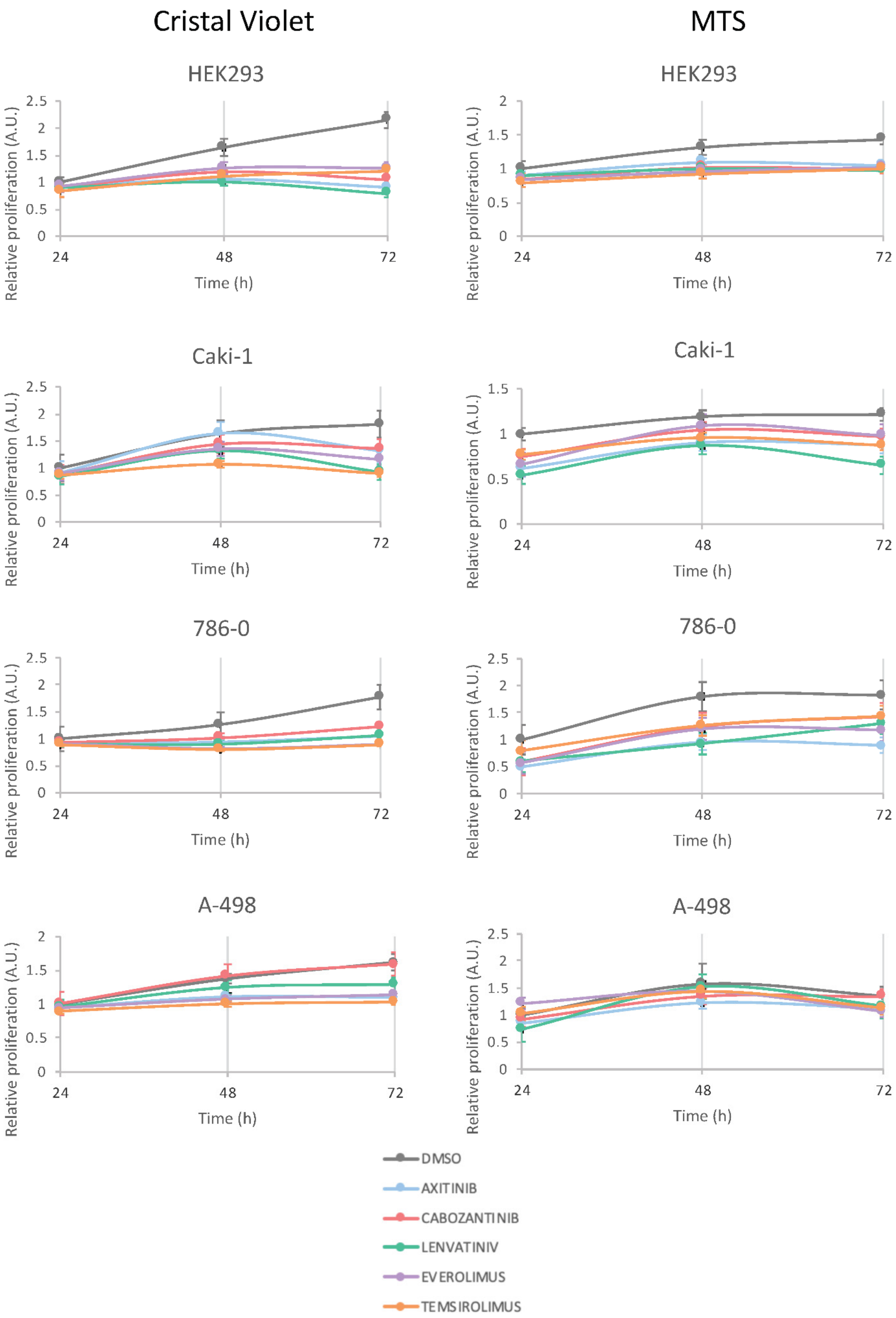

Supplementary Figure S2

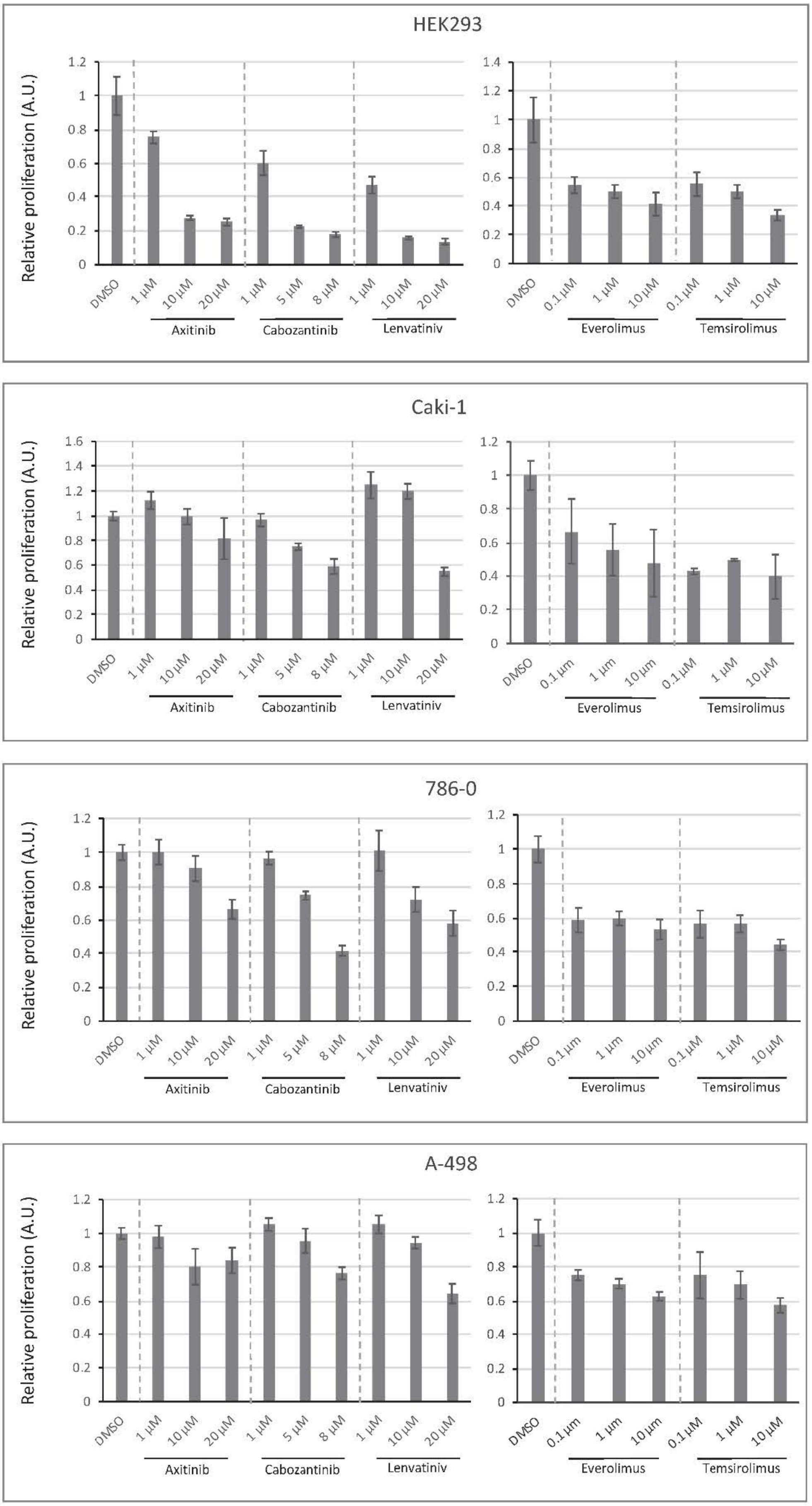

Supplement: Supplementary file 1 [file cells-11-01448-s001.zip › cells-1648526-supplementary.pdf]
